# Is Dignity Still Necessary in Health Care? From Definition to Recognition of Human Dignity

**DOI:** 10.1007/s10943-023-01995-1

**Published:** 2024-01-13

**Authors:** Marcin Paweł Ferdynus

**Affiliations:** Department of Ethics, Faculty of Philosophy, The John Paul II Catholic University of Lublin, Al. Racławickie 14, Lublin, 20-950 Poland

**Keywords:** Dignity, Typologies of dignity, Speciesism, Ecumenical model of dignity, Moral experience

## Abstract

The concept of dignity is not, as some scholars claim, an unnecessary moral idea, and nor need it have religious overtones or be characterised by speciesism. In this article, I try to show that dignity can be defined and recognised. The starting point for the argumentation is the four typologies of dignity, which show that the term ‘dignity’ can denote significantly different concepts, and that the different concepts of dignity can have significantly different ontological senses. A unified typology of dignity allows for five categories to be distinguished: inherent dignity, dignity based on changeable qualities, moral dignity, bestowed dignity and comportment dignity. I take the first two categories of dignity as the object of the analysis, with which I seek to formulate a philosophical response to the charge of speciesism and to show on what basis it can be maintained that all human beings possess dignity. To this end, I distinguish between existential dignity, actual dignity, and potential dignity. Distinguishing these types of dignity becomes possible in the light of Aquinas’ and Aristotle’s views. In the final section, I point to two ways of recognising dignity. The first is based on certain narratives and emotional states (‘ecumenical model of dignity’), while the second is related to a specific moral experience developed within ethical personalism.

## Introduction

The discussion around human dignity is led by representatives of different professions. The issue of dignity is of interest to nurses, doctors, sociologists, psychologists, lawyers, theologians, philosophers and ethicists. Because of this interdisciplinary approach, the concept of dignity has produced a rich literature (Gadow, 1984; Statman, 2000; Chochinov, 2002; Resnik, 2007; Galvin & Todres, 2015; Andorno, 2007; Antiel et al., 2012; Chambers et al., 2014; Fuseini et al., 2022; Franco et al., 2021; Pols, 2013; Waldron, 2012; Dobrowolska, 2010; Chłodna-Błach, 2020; Hughes, 2011; Upenieks, 2022; Bradshaw et al., 2022). The most significant references to human dignity appear in international human rights charters, such as the Universal Declaration of Human Rights, the International Covenant on Economic, Social and Cultural Rights, the International Covenant on Civil and Political Rights, the Convention on the Rights of the Child and the Declaration on the Elimination of Discrimination against Women. Explicit references to human dignity as a value underlying the practice of medicine are contained in other documents: the Declaration of Helsinki, the World Medical Association International Code of Medical Ethics and the Universal Declaration on Bioethics and Human Rights. The importance of dignity in medical practice is also evidenced by various codes of ethics (e.g., the Code of Ethics of Nursing).

In the contemporary discussion around dignity, however, there have been many critical voices. Some scholars think that the concept of dignity lacks clarity (Ferdynus, 2022, p. 351), that it is an unnecessary moral idea, that it has religious overtones and that it is characterised by speciesism (Hoffmann, 2020, p. 603). In his article ‘The stupidity of dignity’, Steven Pinker argues that the principle of respect for the autonomy of the human person provides a sufficient basis in bioethical case law to defend its various values. In his view, it is not necessary to refer to the concept of dignity to justify the need to treat a patient with dignity, especially as this concept is not clear for at least three reasons. Firstly, the concept of dignity is related to the place, time and person it concerns in a given context. Secondly, dignity can be treated interchangeably with other values. Thirdly, invoking the dignity of some people may involve harming others. These reasons lead Pinker to conclude that the concept of dignity is essentially useless (Pinker, 2008). A similar view of dignity is expressed by Ruth Macklin in her article ‘Dignity is a useless concept’. Macklin emphasises that the term ‘dignity’ ‘seems to have no meaning beyond what is implied by the principle of medical ethics, respect for persons: the need to obtain voluntary, informed consent; the requirement to protect confidentiality; and the need to avoid discrimination and abusive practices’. Furthermore, Macklin suggests that dignity ‘is nothing more than a capacity for rational thought and action, the central features conveyed in the principle of respect for autonomy’. Ultimately, Macklin argues that dignity ‘is a useless concept in medical ethics and can be eliminated without any loss of content’ (Macklin, 2003). Thus, both Pinker and Macklin reduce the concept of dignity to the concept of autonomy.

Not all scholars agree with eliminating the concept of dignity from scientific discourse or reducing it to autonomy, however. For example, Hofmann argues that ‘the death of dignity seems to be greatly exaggerated’ (Hoffmann, 2020, p. 610), and Killmister points out that ‘Macklin’s assessment of dignity as a useless concept was premature’ (Killmister, 2010, p. 164). I agree with these views. The proposal to replace the notion of dignity by the notion of autonomy does not yet prejudge anything, because, firstly, the notion of autonomy does not have a single meaning and, secondly, the question of the basis of human autonomy is still open (Bronk, 2010). I believe that there is still a place for dignity in both medical theory and practice. However, a proper distinction between the different meanings of dignity is necessary to avoid misinterpretation.

In this article, I try to show that dignity can be defined and recognised. I first refer to the three strategies by which the claim that human beings are entitled to dignity is justified. I then present four typologies of dignity that show that the word ‘dignity’ can be assigned to significantly different concepts of dignity and that the different concepts of dignity can have significantly different ontological meanings. A unified typology of dignity allows five categories to be distinguished: inherent dignity, dignity based on changeable qualities, moral dignity, bestowed dignity and comportment dignity. I take the first two categories of dignity as the object of analysis, with which I seek to formulate a philosophical response to the charge of speciesism and to show on what basis it can be maintained that all human beings possess dignity. To this end, I distinguish between existential dignity and actual and potential dignity. Distinguishing these types of dignity becomes possible in the light of Aquinas’ and Aristotle’s views. In the final section, I point to two ways of recognising dignity. The first is based on certain narratives and emotional states (‘ecumenical model of dignity’), while the second is related to a specific moral experience developed within ethical personalism.

This paper uses a hermeneutic method that attempts not only to show that the term ‘dignity’ can denote significantly different concepts of dignity and that the different concepts of dignity can have significantly different ontological senses but also to relate them to current discussions and challenges in healthcare ethics.

## Three Strategies for Seeking Dignity

Authors who discuss dignity often have divergent views on what determines or justifies human worth and what moral imperatives follow from the recognition of such worth (Kuhse, 2000; McMahan, 2002; Lee & George, 2008; Dworkin, 2013; Sulmasy, 2013; Rosen, 2018). Different opinions about dignity are associated with different strategies for seeking it. Bronk suggests that three basic, non-exclusive strategies can be distinguished to justify the claim that human beings are entitled to dignity. The first strategy appeals to religious beliefs, the second relates to empirical or a priori philosophical arguments and the third appeals to historical-pragmatic reasons (Bronk, 2010).

Arguments for the ontic dignity of human beings were originally sought on religious grounds (the first strategy). It was recognised that man is a being created in the image and likeness of God. In Catholic theology, for example, human dignity is divided into three levels: (a) the inherent (natural) level of being a person, which ‘by nature’ is common to all human beings (believers and non-believers) and is linked to the fact of being human (i.e., a being endowed with a rational nature and free will); (b) the supernatural level involves being a child of God; and (c) the level of dignity enjoyed by Christians who are in a state of sanctifying grace. The weakness of theological argumentation is mainly that it is only valid for those who appeal to religious assumptions. Those who do not accept the religious rationale either look elsewhere for the basis of dignity or reject dignity as so conceived (Bronk, 2010, pp. 91–92).

Philosophers usually seek the basis for the dignity thesis in a metaphysical conception of the human being (second strategy). In defending human dignity, philosophers refer either to natural law understood as the totality of objective norms arising from human nature or to human subjectivity, freedom and autonomy. An important role in ethical argumentation is played by the well-known postulate that human beings should never be regarded merely as a means but always as an end, as Kant stresses (Kant, 2006). The rationale behind dignity is also the recognition of non-determination and human freedom in making choices (Bronk, 2010, pp. 93–94). However, the fundamental philosophical problem concerning dignity involves trying to answer the question of what a human being is (Gallagher, 2004, p. 592). A weakness of the philosophical strategy is that opinions on rationality and human freedom are the subject of much dispute, and not only among philosophers. As empirical science (e.g. psychology) shows, humans do not always behave as rational beings (Skinner, 1987). Although there are many philosophical theories of the human being, almost every form of philosophical anthropology has been criticised (Ferdynus, 2021).

The historical-pragmatic strategy derives the normative content of the concept of human dignity from the fact that this dignity has been derogated from in the past (third strategy). The historical experience of the instrumentalisation of human beings under totalitarian systems meant that, after the Second World War, the thesis of human dignity was adopted in many national and international documents (Bronk, 2010, pp. 94–95). For example, the Universal Declaration of Human Rights opens with the words ‘Whereas recognition of the inherent dignity and of the equal and inalienable rights of all members of the human family is the foundation of freedom, justice and peace in the world’ (Universal Declaration of Human Rights, 1948). The basis for human dignity is also sought in the social contract, in statute law and in prevailing social and cultural customs.

Each dignity typology, created by different authors, refers to one, two or even three of the strategies described. Below, I will describe four typologies that are discussed in the literature.

## Typologies of Dignity

In this section, I present typologies of dignity according to Seifert, Nordenfelt, Schroeder and Piechowiak. These typologies of dignity show that the word ‘dignity’ can denote significantly different notions of dignity. In a unifying typology of dignity, I distinguish five categories: inherent dignity, dignity based on changeable qualities, moral dignity, bestowed dignity and comportment dignity.

### Seifert’s Typology of Dignity

Within personalism, the German philosopher Seifert distinguishes four concepts of dignity: ontological dignity, dignity of the conscious subject, moral dignity, and dignity as a gift (Seifert, 2004, 2013).

Man has ontological dignity from the moment of the existence of human nature. The ontological dignity of the person belongs to all existing human beings, whether they are currently conscious, healthy or ill, or born or developing in the prenatal period. This dignity excludes the subjective or utilitarian treatment of human beings and is the basis of the right to life and its development. It is not given to a person ‘from the outside’, but is found with their existence. This dignity is not gradable; all people possess it equally. It is not determined by contract, consensus or arbitrary legislation. During a person’s life, ontological dignity is immutable and cannot be taken away. It has absolute, ontic and temporal priority over the other three types of dignity (Seifert, 2013, pp. 16–17).

The dignity of the conscious subject is revealed in the conscious actualisation of rationality, freedom and love. This dignity is revealed in intentional acts of cognition, in language, in knowledge and in conscious and responsible relating to other beings. This type of dignity does not have the characteristics of fixity and immutability. It does not occur during prenatal development and may be more or less limited by injury (e.g. comatose state, persistent vegetative state). A person can develop this dignity but can also easily lose it. The dignity of the conscious subject is the basis for various rights (e.g. the right to knowledge, the right to truth, the right to hold one’s own worldview, the right to religious freedom, the right to freedom of conscience, the right to love) (Seifert, 2013, pp. 17–18).

Moral dignity is achieved through the proper actualisation of the human personality. Humans perfect themselves through rational and free acts. Moral dignity is acquired through good acts, in particular through the development of fixed skills called moral virtues. Moral dignity is not given to humans with existence, but is shaped by appropriate moral conduct – that is, by acting in accordance with the truth. This dignity can be lost through bad acts and through the development of fixed qualities called vices. Moral dignity is gradable, so it is possible to have low or minimal moral dignity. A person can also develop a personality contrary to moral dignity – that is, evil, vile and unworthy (Seifert, 2004, pp. 126–128).

Dignity as a gift involves the relationship of the person to other persons, to the community and to God. This type of dignity is bestowed on a person from outside, by other people (the human community) or by God. It is a gift that takes into account the natural, interpersonal relationships formed in a family or an ethnic, national or religious community. This dignity is associated with a person’s different abilities and talents, but is more than these abilities or talents. In addition, various forms of dignity that are conferred by social and state institutions grow out of social relationships. The attribution of important professional, social, political functions to individuals gives them dignity and authority (Seifert, 2004, pp. 128–130).

### Nordenfelt’s Typology of Dignity

Nordenfelt presents four types of dignity: the dignity of merit, the dignity of moral stature, the dignity of identity and universal human dignity (German *Menschenwürde*) (Nordenfelt, 2004).

Dignity of merit is linked to the performance of certain roles, functions or offices. For example, a king, a minister, a bishop or a doctor have a special dignity by virtue of their position. These are formal dignities of merit. Usually, these merits are conferred on people by a formal act (e.g., an appointment). In some cases, a person may be born with such dignity (the case of hereditary monarchy). Dignity of merit is linked to notions of rights and respect. By virtue of their position, a person may have special rights. What matters is that ‘the dignities of merit can come and go. People can be promoted but they can also be demoted. People can for some time have an informal fame and high reputation, but this can suddenly be gone. Another feature of the dignities of merit is that they admit of degrees’ (Nordenfelt, 2004, p. 72).

Dignity as a moral stature depends to a large extent on a person’s thoughts and actions. It is linked to the idea of a dignified character and dignity as a virtue. In other words, this type of dignity is based on an individual moral attitude that is revealed through a person’s actions. Nordenfelt notes that there is ‘an important difference between the ordinary dignities of merit and the dignity of moral stature in that the latter does not provide the subject with any rights’. Moreover, he suggests that respect is related to moral dignity in several ways: (a) humans are inclined to show respect to others; (b) there is a particular respect that a human deserves, but this respect is not linked to any of their rights; and (c) the human can show respect to themselves. Like dignity of merit, dignity as a moral stature has different degrees. It can be diminished or lost by immoral acts (Nordenfelt, 2004, pp. 73–74).

Dignity of identity is the type of dignity attributed to the human being as an integrated and autonomous person, a person with their own history and with all the relationships they form with other human beings. Nordenfelt grants the dignity of identity an objective status and bases it on the integrity and autonomy of the subject. He writes, ‘[T]he facts that ground the dignity of identity are the subject’s integrity and autonomy, including his or her social relations. These facts are typically associated with a sense of integrity and autonomy. And when a person’s integrity and autonomy are tampered with this is typically associated with a feeling of humiliation or loss of self-respect on his or her part’ (Nordenfelt, 2004, p. 76). Dignity of identity is therefore the kind of dignity that can be taken away from a person by external events, the acts of other people, as well as by injuries, illness and old age. Someone ‘from the outside’ can invade a person’s private sphere, hurt them or limit their autonomy. Nordenfelt describes these acts as follows: ‘Intrusion in the private sphere is a violation of the person’s integrity. Hurting a person is not only violation of integrity; it also entails a change in the person’s identity. The person is after this a person with a trauma; he or she has in a salient sense a new physical identity. The person’s autonomy can be tampered with, when the person is prevented from doing what he or she wants to or is entitled to do. Finally, insulting, hurting or hindering somebody entails excluding this person from one’s community’ (Nordenfelt, 2004, p. 76). According to Nordenfelt, the assumption of the objective nature of identity helps to explain why the dignity of the unconscious and even the dignity of the dead can be said to be violated (Nordenfelt, 2004, p. 77).

The universal human dignity denoted by the German word *Menschenwürde* refers to a certain kind of dignity that people have simply because they are human. It is a specifically human value. Nordenfelt writes about this value as follows: ‘Menschenwürde is a dignity belonging to every human being to the same degree all through his or her life. It cannot be taken away from any person and it cannot be attributed to any creature by fiat. The dignity of Menschenwürde is the ground for the specifically human rights’ (Nordenfelt, 2004, p. 79). He also points to several features that distinguish *Menschenwürde* from other types of dignity, especially from the dignity of identity. *Menschenwürde* ‘is once and for all fixed and it is the same for all people’; it ‘can *ex hypothesi* not get lost’ and ‘is *ex hypothesi* tied to the living human being’. Nordenfelt points to two bases for the recognition of universal human dignity: (a) the Christian tradition (humans are created in the image of God) and (b) the philosophical tradition (reference to key human capacities such as consciousness, freedom, autonomy) (Nordenfelt, 2004, pp. 78–80).

### Schroeder’s Typology of Dignity

Like Seifert and Nordenfelt, Schroeder identifies four types of dignity: Kantian dignity, aristocratic dignity, comportment dignity and meritorious dignity (Schroeder, 2008).

In his classic text *Groundwork for the Metaphysics of Morals*, Kant characterises dignity as an intrinsic value (Kant, 2006, 4:434), unconditional, incomparable and respectable (Kant, 2006, 4:436). What dignity possesses is priceless, irreplaceable and not exchangeable for anything else. Kant writes about dignity as follows:

“In the kingdom of ends everything has either a price or a dignity (Würde). What has a price can be replaced by something else as its equivalent; what on the other hand is raised above all price and therefore admits of no equivalent has a dignity. What is related to general human inclinations and needs has a market price…but that which constitutes the condition under which alone something can be an end in itself has not merely relative worth, that is, a price, but an inner worth, that is dignity…Hence morality, and humanity insofar as it is capable of morality, is that which alone has dignity.” (Kant, 2006, 4:434–435).

Drawing on Kant’s views, Schroeder notes that human beings have dignity because of their rational nature, which is capable of moral self-determination. Schroeder emphasises that ‘a person with dignity has rights, an absolute inner worth by which he extorts respect for himself from all other rational beings in the world’ (Schroeder, 2008, pp. 232–233). Thus, it can be said that someone who has rights, who can legislate for themselves, has dignity. Dignity, on the other hand, does not allow a person to be treated instrumentally. Kant expresses this thought in the ‘Formula of Humanity’: ‘So act that you use humanity, whether in your own person or in the person of any other, always at the same time as an end, never merely as a means.’ (Kant, 2006, 4:429). Inspired by Kant, Schroeder defines Kantian dignity as follows: ‘Dignity is an inviolable property of all human beings, which gives the possessor the right never to be treated simply as a means, but always at the same time as an end’ (Schroeder, 2008, p. 233).

Schroeder refers to the second type of dignity as aristocratic dignity. She points out that the Latin term *dignitas* means ‘ornament, distinction, or glory’. In pre-modern times, dignity was associated with rank rather than a universal attribute of the human race. In stratified societies, reference was made to dignity to emphasise that some people were valued more highly than others. Dignity bearers held high secular and religious positions. Being dignified meant, foremost, acting in accordance with the requirements of one’s position. Aristocratic dignity was therefore limited to a small number of people in society and closely linked to social position. Schroeder defines aristocratic dignity as ‘the outwardly displayed quality of a human being who acts in accordance with her superior rank and position’ (Schroeder, 2008, p. 233).

The third type of dignity Schroeder calls comportment dignity. This type of dignity is similar to aristocratic dignity in the sense that it concerns the outward manifestations of proper behaviour. However, it differs from the second type in that the determining factor for appropriate behaviour is not rank and position, but adherence to social norms and expectations. Comportment dignity is most easily captured in a negative sense. It may, for example, be undignified to tell ‘a rude joke at an official dinner with one’s mouth full, to giggle at an obituary’, ‘to spit onto the street, to undress or relieve oneself in public’. This type of dignity is defined by Schroeder as ‘the outwardly displayed quality of a human being who acts in accordance with society’s expectations of well-mannered demeanour and bearing’ (Schroeder, 2008, p. 234).

The final type of dignity Schroeder refers to as meritorious dignity. Referring to Aristotle’s views, she points to two aspects of meritorious dignity. First, ‘[d]ignity consists in deserving not displaying honours, in other words, being honourable’. Dignity is combined with the virtue of temperance, courage, justice and wisdom. Second, dignity is revealed in facing adversity. To cope with unhappy situations in life, one needs inner strength, which is based on the cardinal virtues, as well as self-worth. Those who excel in temperance, courage, justice and wisdom have dignity. Schroeder defines meritorious dignity as ‘a virtue, which subsumes the four cardinal virtues and one’s sense of self-worth’ (Schroeder, 2008, p. 235).

### Piechowiak’s Typology of Dignity

The typology of dignity proposed by Piechowiak differs from previous typologies in that he distinguishes certain meanings of ‘dignity’ based on an analysis of national and international law documents. Piechowiak identifies six types of dignity as properties of the person: inherent dignity of the person, dignity based on observable and changeable characteristics specific to a rational being, dignity based on historically formed social status, personal dignity, dignity as moral excellence and dignity as appropriateness of behaviour (Piechowiak, 2022).

The inherent dignity of the person has several characteristics. This dignity is innate, inalienable, equal, universal and inviolable. The innate nature of dignity means that it is not acquired through anyone’s actions, from the circumstances of life or by virtue of possessing any variable characteristics. The inalienability of dignity means that it is not lost either as a result of anyone’s actions or due to the acquisition or loss of any variable quality. Another feature – equality – means that every human being has equal dignity. It is non-gradable and cannot be higher or lower. The universal nature of dignity means that it is enjoyed by all human beings. The inviolable nature of dignity means that it cannot be ‘depleted’ of something and cannot be ‘diminished’ in any way. Conditions, states of affairs due to a person may be violated, but their dignity cannot be violated. According to Piechowiak, the first four characteristics of dignity should be linked to the ontological (metaphysical) status of the person. This means that the inherent dignity of the person goes beyond what is ‘physical’. It cannot be equated with any observable human characteristic. The inherent dignity of the person cannot, therefore, be the direct object of study of the specific sciences, such as physics, chemistry or biology (Piechowiak, 2022, p. 13). According to Piechowiak, the final feature – inviolability – should be linked to situations where personal dignity clashes with other values. He suggests that when such a collision occurs, it is not permissible to compare personal dignity with other values, because the inherent dignity of the person is incomparable (Piechowiak, 2022, p. 14). Inviolability thus turns out to be a quality that prohibits purely instrumental treatment of human beings and dictates that human beings must always be treated as an end in themselves. Referring to Kant, Piechowiak emphasises that ‘dignity is unconditional, it is an absolute value in the sense that it is not permissible to recognise any hypothetical imperative with a structure: *if such and such a value requires protection, one can act against dignity*. Dignity is the basis of the categorical imperative that allows for no *if*’ (Piechowiak, 2022, p. 15).

The second type of dignity is one based on observable and changeable characteristics specific to human beings. In contemporary philosophical discussion, certain human qualities (e.g., self-consciousness, the ability to think, to make free choices, to plan for the future) are recognised as the basis for being a person and thus for having rights. One prominent representative who advocates this way of thinking about the basis of being human (person) is Singer (2011). In this view, a human is determined to be a person if they possess the relevant qualities (e.g., self-consciousness). However, if a human does not have the appropriate qualities, they are not a person. Thus, dignity based on variable characteristics can be greater or lesser, acquired or lost. According to Piechowiak, this type of dignity is not an adequate concept for human rights settlements in international law (Piechowiak, 2022, pp. 17–18).

The third type of dignity is the dignity associated with social status. In this view, dignity is based on institutionalised social constructs that determine a particular social status related to the possession of rights. Historically, it is possible to point to examples when full rights were granted only to free people or citizens. This way of understanding dignity as a basis for rights is rejected today. Basing the fullness of rights on institutionalised social constructs can exclude some people from the circle of subjects. In Piechowiak’s view, this type of conception of dignity is not consistent with the settlements adopted in the legal protection of human rights, in particular the recognition that dignity is innate and inalienable (Piechowiak, 2022, p. 18).

Personal dignity, the fourth type of dignity, involves an internal aspect (self-worth) and an external aspect (good reputation, honour). This type of dignity is fundamentally dependent on the actions of other people. The basis of personal dignity is the individual’s idea of themselves and of their own worth in the social roles they perform (internal aspect). Linked to the self-worth perspective is the expectation of respect from other people (external aspect). Personal dignity can be violated from the outside, for example when someone, contrary to their own beliefs about a certain person, speaks in public in such a way as to take away that person’s good name. While the proper attitude towards personal dignity is respect towards a person, insult or contempt takes away a person’s dignity (Piechowiak, 2022, p. 20).

The fifth type of dignity is the moral excellence of the subject. Piechowiak regards Ossowska’s opinion as the classic definition of this kind of dignity: ‘[D]ignity is manifested by those who know to defend certain values, recognised by themselves, when with the defence of these values the sense of their own value is connected’ (Ossowska, 1969). Piechowiak stresses that this type of dignity is about being faithful to one’s conscience, about not doing what one has promised oneself not to do. Furthermore, he maintains that only the subject can violate this dignity and thus become morally better or worse. Other subjects can induce or coerce the subject to act contrary to moral convictions, but they cannot make the subject morally good or bad (Piechowiak, 2022, p. 49).

The final type of dignity is dignity as appropriateness of behaviour. Aristotle describes this kind of dignity in the *Eudemian Ethics* (Aristotle, 1992, 1221a). Wainwright and Gallagher note that dignity as described by Aristotle is determined as ‘one of 14 virtues or mean states of character, between an excess of unaccommodatingness and of deficiency of servility’ (Wainwright & Gallagher, 2008, p. 47). It can be smaller or larger; it can be acquired or lost. Piechowiak emphasises that this type of dignity essentially concerns actions that are not so much unacceptable as inappropriate (Piechowiak, 2022, p. 22).

### A Unified Typology of Dignity

The typologies of dignity described above show that the term ‘dignity’ can be assigned to substantially different concepts of dignity and that the different concepts of dignity can have substantially different ontological statuses. ‘Dignity’ can mean something innate or intrinsic (inherent dignity), a characteristic or set of characteristics (dignity based on changeable qualities), acquired moral qualities (moral dignity), acquired relational qualities (bestowed dignity) or an attitude or behaviour (comportment dignity). One possible way of ranking the described typologies of dignity is shown in Table 1.

**Table 1:** Unified typology of dignity – own elaboration

Types of Dignity					
	inherent dignity	dignity based on changeable qualities	moral dignity	bestowed dignity	comportment dignity
Seifert	+ (ontological dignity)	+ (dignity of the conscious subject)	+ (moral dignity)	+ (dignity as gift)	
Nordenfelt	+ (Menschenwürde)	+ (dignity of identity)	+ (dignity of moral stature)	+ (dignity of merit)	
Schroeder	+ (Kantian dignity)		+ (meritorious dignity)	+ (aristocratic dignity)	+ (comportment dignity)
Piechowiak	+ (inherent dignity of the person)	+ (dignity based on changeable qualities)	+ (dignity as moral excellence, personal dignity)	+ (dignity based on social status)	+ (dignity as appropriateness of behaviour)

In the typology of meanings proposed here, it is accepted that the boundaries between the types may be blurred (they may sometimes overlap), that the criteria for belonging to a given type may to some extent appear in the characteristics of other types and that not all understandings of the term ‘dignity’ are included. There is a rich literature on various aspects of dignity (Leget, 2013; Jacobson, 2009; Van Der Graaf & Van Delden, 2009; Jacelon et al., 2004; Schroeder, 2010). The aim of the reflection carried out here is not to produce a comprehensive elaboration of the different types of dignity, but to outline a map that can be used to identify the different meanings ascribed to the term ‘dignity’ in bioethical debates. Considering the typology of dignity presented, one can see, for example, that proponents of euthanasia, abortion or assisted suicide most often refer to dignity based on changeable qualities, whereas opponents of euthanasia, abortion or assisted suicide refer to inherent dignity. Similarly, it may be thought that those who refer to dignity based on changeable qualities recognise that patients in a persistent vegetative state (PVS) have lost their normative status. In contrast, those who accept the inherent dignity view recognise that patients in a PVS still retain normative status – that is, they have dignity. It seems that the key dispute concerns precisely these two types of dignity: dignity based on changeable qualities and inherent dignity. Those who defend inherent dignity usually refer to Kant. However, it seems that Kant is not the best candidate to defend inherent dignity in the bioethical debate. Kantian dignity cannot fully answer the charge of speciesism (by ‘speciesism’ I mean here the view that not all human beings have superior status to nonhuman beings) (Singer, 2009, pp. 573–574). In the next section, I will try to show what arguments can be used by those who recognise that, regardless of the quality of the biological condition (e.g., the loss of certain qualities), every human person has inherent dignity for as long as they exist.

## A Response to the Charge of Speciesism

Kant’s key views on dignity are contained in the already cited publication *Groundwork for the Metaphysics of Morals*. Kant argues that a human is a person because they possess dignity, that they are an end in themselves (Kant, 2006, 4:428). He regards the principle of autonomy as the most important principle of morality. For Kant, autonomy is the basis of the dignity of nature and every rational nature (Kant, 2006, 4:436). It should be emphasised, however, that autonomy, although it ‘involves making a law for oneself, does not involve freely disposing of the content of that law – on the contrary, it presupposes that it is a universal law whose content can be known and which one does not freely shape oneself’ (Piechowiak, 2011, p. 15). The basis, therefore, of the dignity of a rational being is participation in universal law. Furthemore, Kant perceives the individual human being as a specimen of the species in the sense that proper action is uniquely determined by what is general. Action should be determined according to universal laws (Kant, 2006, 4:421). In other words, the condition for a particular person to achieve their proper perfection is to act according to what is universal and not according to what is individual. From this perspective, Kant’s formula of humanity plays an important role. For Kant, the common humanity of all people constitutes the foundation of dignity and person (Piechowiak, 2011, p. 17). Thus, if one considers that a person is merely a specimen of the species *Homo sapiens*, it is impossible to avoid the charge of speciesism. Moreover, if one considers that human beings possess dignity because ‘they are rational, autonomous creatures with intrinsic value who can pursue and determine their own ends’ (Badcott, 2003, p. 124), one must at the same time agree that Kantian dignity seems to be limited only to those persons who possess certain characteristics such as rationality and autonomy (Wainwright & Gallagher, 2008, p. 47). The basis of dignity for Kant is not simply being human, but ‘having the power of rational agency’ (Zylberman, 2016, p. 205). Thus, the absence of key qualities in some people (e.g., PVS, severely disabled, comatose) does not allow one to attribute dignity to them in the Kantian sense. In looking for arguments to answer the charge of speciesism and to justify why all human beings have dignity, one must turn to the views of Thomas Aquinas and Aristotle.

### Existential Dignity

Aquinas considers dignity to be the fundamental perfection of man that distinguishes him from other beings (things). He believes, as does Kant, that dignity is a constitutive property of the person. This means that one is a person because one has dignity, not the other way around. In his *Commentary on the Sentences of Peter Lombard*, Aquinas says that ‘the name *person* signifies an individual substance as having a propriety which is a sign of dignity’ (Thomas Aquinas, 1996, *Scriptum super Sententiis*, lib. I, d. 23, q. 1, a. 1 co). In turn, in the *Summa Theologiae*, he adds, ‘because subsistence in a rational nature is of high dignity, therefore every individual of the rational nature is called a *person*’ (Thomas Aquinas, 1996, *Summa Theologiae* (STh), I, q. 29, a. 3, ad 2). Commenting on the passages quoted here, Piechowiak notes that ‘one is entitled to the name *person* because of dignity, and dignity is grounded not in rationality itself, as a property of man, but in existing in a particularly perfect way which is specific for intelligent beings. When Aquinas characterises a person, he does not talk directly about an individual of a certain kind (of a certain nature), but about subsistence (Latin: *subsistens*) in a certain nature. It could be said that rationality is primarily a feature that makes it possible to decide who is entitled to the name *person* (it is a diagnostic property), but it is not the real reason for calling someone a *person*’ (Piechowiak, 2016, p. 72). Thus, for Aquinas, rationality is not a perfection of the human being that alone constitutes the person (ontological perspective). In other words, rationality is not that perfection by virtue of which one calls something a person (Piechowiak, 2011, pp. 6–7). To define what dignity is, in the sense of the perfection that constitutes the person, it is necessary to refer to Aquinas’ views, which are contained in Article 1 Question 29 Part 1 of the *Summa Theologiae* (STh, I, q. 29, a. 1, co). Drawing on Aquinas’s views, Piechowiak writes, ‘The reason for naming a being a person is that the particular and the individual (*particulare et individuum*) are found in this being in a more special and perfect way (*specialiori et perfectiori modo*). According to Aquinas, a more special and more perfect particularity and individuality prove the perfection of a being’s existence’ (Piechowiak, 2016, p. 72). What is more individual is more unity (*unum*), and the more particular (*particulare*), the more distinct (*aliquid*) (Piechowiak, 2011, p. 7). In *De Potentia*, Aquinas defines the particular unity of the person as individuality (*individualitas*), while the special particularity of the person as incommunicability (Thomas Aquinas, 1996, *De potentia*, q. 9, a. 6, co). Thus, ‘the more something is a unity and the more something is a particularity, the stronger it exists’ (Piechowiak, 2016, p. 73). If, therefore, the foundation of the status of the person, which constitutes the greatest inherent perfection of the human being and distinguishes them from other beings, is sought, the focus must be on the existential endowment of being and not on the ontological endowment (content). The rationality belonging to the content endowment is a fundamental perfection, but it is not the greatest perfection. Excellence at its greatest is dignity understood as a particularly strong mode of existence, a particular individuality and distinctiveness, existence as an end in itself (Piechowiak, 2011, p. 9). If one additionally refers to the place where Aquinas considers the permissibility of the death penalty, one can point to two properties that characterise human beings’ mode of existence: being free and existence for its own sake (*homo est naturaliter liber et propter seipsum existens*) (STh, II-II, q. 64, a. 2, ad 3). These two properties indicate that human beings exist as an end in themselves (Piechowiak, 2011, p. 9; Piechowiak, 2016, p. 77). The key issue for the analyses conducted here is the phrase ‘existence for its own sake’. This phrase was defined by Aquinas in the *Summa Contra Gentiles* (Thomas Aquinas, 1996, *Summa contra Gentiles*, 3, cap. 113). Analysing Aquinas’s thought, Piechowiak states that ‘[h]ere Aquinas justifies the thesis that the aim of intelligent beings is to constitute themselves not only by what is generic, as is the case with animals, but also according to what is individual; in contrast to animals, individual people are not just specimens of the human race’. He also adds, ‘The aims pursued in the here-and-now by man (it should be emphasised – aims leading to man’s development) are not generic (not determined entirely by the human nature shared by all human beings), but individual. Indeed, an aim which involves development, happiness or salvation is specified, as is the need to develop the possessed dispositions. However, the manner of this development is not specified’ (Piechowiak, 2016, p. 78).

In summary, it can be said that, for Aquinas, the foundation of being a person is dignity understood as existing in some particularly perfect way. The perfection of a person’s existence is expressed through individuality and distinctiveness. Human beings are more individual and distinct beings than other beings by virtue of the fact that their perfection – existence for its own sake – is grounded in freedom. Thus, human beings can take action and develop individual dispositions that are not clearly determined by universal law (as in Kant). Moreover, dignity understood as a particular mode of existence encompasses the human being in all its dimensions, in what is related to self-consciousness as well as in what is corporeal (Piechowiak, 2011, p. 20). Therefore, as long as a human being exists, they have a dignity proper to themselves, regardless of changing qualities. Hence, this can be called the existential dignity.

It seems that the conception of dignity presented here can answer the charge of speciesism. Firstly, existential dignity points to the unique character of each human person, which is expressed in a unique and incommunicable act of existence. Secondly, existential dignity provides a rationale for why the human being cannot be seen merely as a specimen of the species, because the human being transcends species conditioning. It should also be emphasised that existential dignity provides a philosophical answer to the question of why all human beings have dignity.

### Actual and Potential Dignity

The observable and changeable qualities attributed to rational beings are often the basis for granting or denying dignity to someone. If someone has the right qualities (e.g., self-consciousness), they have dignity; if they do not have these qualities, they do not have dignity. When looking for arguments to justify why the loss of qualities is not sufficient grounds for denying dignity, one should turn to Aristotle’s views, especially the theory of act and potency.

In the *Metaphysics*, Aristotle included the classical formulation of act (Lat. *actus*): ‘Act is what belongs to a thing, but not as it [a thing] is understood in potency’ (Arystoteles [Aristotle], 2017, 1048 a 31). An act is a mode of being of a thing that is different from potential being. It is the completed realisation or the process of the realisation of potential being. Aristotle explains: ‘[T]hat which is actually building is to that which is capable of building so is that which is awake to that which is asleep; and that which is seeing to that which has the eyes shut, but has the power of sight; and that which is differentiated out of matter to the matter; and the finished article to the raw material. Let actuality be defined by one member of this antithesis, and the potential by the other’ (Aristotle, 1980, 1048 b 1–10).

Potency (Lat. *potentia*), on the other hand, means a subject’s disposition or the foundation of changes. The determination of the potency is revealed in close connection with the act. Hence, the kind of act is like the potency – that is, potency as the disposition of a being to receive action and potency as the ability of a being to act. According to Aristotle, all composite beings possess a state of possibility and actuality. This means that every being consists of a potency and an act. Moreover, each of these factors – act and potency – carries with it specific determinations that reveal the nature of things. Maryniarczyk summarises Aristotle’s theory of act and potency as follows: ‘The potency and act that occur in a concrete being always occur in the same category of being, that is, they belong to the same order of being. Hence we learn about potency through the acts that emerge from it, but not the other way around. This allows us to learn about the nature of the acting subject, whether it is a question of a human being or any other acting being’ (Maryniarczyk, 2018, p. 80).

Human beings can therefore be understood in terms of what their potency and their act are. Act and potency always appear as two states of the same being. Through them, it is possible to understand and explain the fact of action, perfection and development of human beings. This process is the actualisation of the potency residing in a person (Maryniarczyk, 2018, p. 91). By referring to the theory of act and potency, it is possible to explain what concerns dignity based on changeable and observable qualities. According to Aristotle’s assumptions, if someone has the actual ability for rational and moral self-determination, this capacity must also reside in the potency, because there is not in the act what is not in the potency. It is possible to know by the actualisation, or expression, of certain abilities that someone had them in their potency, although originally they were as if in a ‘passive’ or ‘dormant’ state. Under relevant conditions of human nature, these capacities are actualised and can be recognised empirically. Thus, the dignity based on the actualisation of capacities residing in the potency I call actual dignity. It is available to the vast majority of people (e.g., the ability for rational and moral self-determination). A few are deprived of it (severely disabled, PVS patients, comatose, unborn, etc.). However, if act and potency are always two states of the same being, then even when human nature is ‘deficient’ or ‘diseased’ (i.e., does not allow for the expression of certain qualities), a human does not therefore become less human. These capacities still reside in the potency, although due to a defect in nature, they cannot temporarily or permanently be expressed. The dignity based on the capacities residing in potency I call potential dignity. From the perspective considered here, it is therefore reasonable to argue that every human being has dignity. Those who have actual dignity also have potential dignity. Those who are prevented by their ‘sick nature’ from expressing qualities (e.g., self-consciousness) have only potential dignity. Such a thesis is legitimate on the assumption that actual and potential dignity are two states of the same being.

Similar intuitions are referred to by Killmister (2010), although she does not mention Aristotle’s theory of act and potency. Killmister suggests that to make a unified sense of the concept of dignity, a distinction must be made between capacity and ability. According to her, capacity is the latent potential, while ability refers to the immediate ability to act. For example, an athlete with an injury may lose the ability to compete, but may not lose capacity. Under the influence of appropriate therapy, a lost skill (ability) can be restored. Killmister states that ‘[t]o see dignity as the capacity for principled action, therefore, is to recognise that there is a latent potential in all persons so to act. Even if events make an instance of virtue impossible – an individual does not have the ability to remain courageous under conditions of torture, for example, or to uphold their standards of personal hygiene in substandard hospital care – their capacity remains intact’ (Killmister, 2010, p. 162). Surprisingly, however, on the one hand, Killmister recognises that all persons have latent capacities, while on the other hand, she expresses doubt that all persons have dignity. For her, the attribution of dignity to all members of *Homo sapiens* is merely a postulate with which to reconcile the theoretical justification of dignity with the practical difficulties faced by medical practitioners (Killmister, 2010, p. 163). However, the solution to this problem seems to be to refer to Aristotle and assume that all people have potential dignity based on latent capacity and that the vast majority of people have actual dignity based on ability. A more radical move is to appeal to Aquinas and recognise that all human beings have an existential dignity, which is the basis for all other types of dignity.

Even if the proposed solution regarding the understanding of dignity is not acceptable to some participants in the ethics debate, one can at least hope for a mutual understanding of the different positions on the issue.

## Recognising Dignity

Dignity can be examined not only in terms of the properties or qualities that people possess, but also from the perspective of describing the human experience. In this section, I discuss two ways of recognising dignity. The first is based on certain narratives and emotional states, while the second is related to a specific moral experience developed within ethical personalism.

### An Ecumenical Model of Dignity

An interesting proposal on the recognition of human dignity is presented by Napier (2020). He suggests that his approach is ecumenical in the sense that it does not focus on a single property or function by which dignity can be defined, but emphasises the irreplaceable value and preciousness of the human person. His approach seeks to discover the dignity of the person in the light of narratives and certain emotional states. Napier’s proposed way of recognising dignity, which can be described as an ecumenical model of dignity, includes three emotional states: remorse, grief and love.

Napier observes that remorse sheds light on the irreplaceable value of the one who has been wronged. A person who experiences remorse begins to understand that they have wronged someone. By feeling remorse, a person realises that ‘others are fundamentally equal to oneself’ (Napier, 2020, p. 89). Napier depicts the nature of this experience in relation to a woman who regrets her abortion. Referring to Johnson and Detrow’s book (2016), he describes the controversial case of a 30-year-old woman, Angie, who decides to have her ninth abortion. Angie seems proud of her indifference to yet another abortion, which embarrasses even the experienced staff at the abortion clinic. After the procedure, Angie asks to be shown the remains, saying, ‘I’ve had it done so many times, I might as well know what it looks like’ (Johnson & Detrow, 2016, p. 74). From the laboratory, the employee brings the ‘product of conception’. When Angie looks at the remains of the abortion, her behaviour changes dramatically. A clinic worker recalls Angie’s experience as follows:

“When her eyes travelled to the container, she gasped sharply, and for the first time since she had arrived, Angie was utterly silent. A few moments later her entire body shuddered and gooseflesh was raised on her smooth brown arms. When she reached out her hand to touch the baby, I tried to pull the dish away. She grabbed my wrist and stopped me. We were both silent for a few moments as she continued to stare at the contents of the dish. I stepped back, Angie fell forward to her knees, her fingers still wrapped around my wrist. The other girls in the recovery room began to take notice, and my discomfort level rose exponentially…She remained frozen on the clinic floor. ‘That’s a baby,’ she said, barely audible at first. ‘That was my baby,’ she said. Her volume steadily increased as a torrent of words poured from her mouth.” (Johnson & Detrow, 2016, p. 75).

Napier notes that remorse made the woman realise that the remains in the dish were her child. Moreover, the remorse showed her how precious her child was. Referring to the role that remorse plays in recognising dignity, Napier states that ‘[t]he preciousness of the individual is lit up by the remorse of having wronged him or her. The argument here is that remorse is a common emotion that is apposite in many cases. If the object of remorse turns out to be the preciousness of the person, remorse functions as a periscope by which one glimpses human worth’ (Napier, 2020, p. 90).

The second emotional state through which to recognise a person’s dignity is grief. Referring to Brewer’s views, Napier notes that mature grief following the death of a loved one involves the knowledge that nothing can provide compensation in return for what has been lost. After the loss of a person, only consolation is possible; no compensation is possible (Napier, 2020, p. 91; Brewer, 2009, p. 174). One does not mourn the loss of a person’s qualities, properties or abilities. A person may be sociable, kind, funny, but one does not mourn the loss of these qualities; one mourns the loss of the person. It can be said that ‘grief is not just a generic pro-attitude towards an irretrievable entity with a certain set of natural properties; grief lights up its lost object as having had a very particular sort of value’ (Brewer, 2009, p. 176). The object of grief cannot be a feature or property of a person, but the person themselves. Grief makes one realise that something of value has been lost. Apt intuitions in this context are expressed by Zagzebski: ‘If someone is irreplaceable in value, I assume that means that if we lose her, no one else, no matter how similar to her, can replace her. That must mean that part of her value comes from something about her that nobody else has’ (Zagzebski, 2001, p. 413). It is therefore right to believe that ‘grief lights up the individual preciousness of the person’ (Napier, 2020, p. 91). To illustrate the grief that allows the dignity of the person to be captured, Napier points to a patient named Joe who is in a state of permanent unconsciousness:

“To be sure, we think it sad and grieve the fact that Joe is in an unconscious state partly because he ought not to be in such a state. We do not mourn the fact that a tulip is not conscious, but that is because of the kinds of things tulips are. We do mourn the fact that Joe is in a permanent unconscious state because of who Joe is, namely a human being. The fact that we mourn that Joe is in a permanently unconscious state indicates that he has suffered an *injury* or an *assault* on who he is by nature. We should resist inferring from our mourning that Joe has no worth at all; my view holds that our mourning is a sign that we are countenancing Joe’s dignity. We do not grieve the absence of consciousness per se, but that *Joe* has lost consciousness. Again, the various states, abilities, and capacities of a person are parasitic on the person. We do not mourn *their* absence but mourn because *the person* is deprived of them.” (Napier, 2020, p. 96).

The final factor with which to capture dignity is love. Napier emphasises that if someone loves someone else, they do not love that person’s qualities, characteristics or abilities, but they love them as a person. To illustrate the moral intuitions associated with love, Napier refers to a story in Gaita’s book *Good and Evil: An Absolute Conception* (2004). Gaita reports the story of Primo Levi, who was imprisoned in Auschwitz. One of the other prisoners, Ladmaker, a 17-year-old Dutch Jew, was suffering from typhoid fever and scarlet fever and also had a bad heart. He was bedridden due to illness and malnutrition. The bedsores meant that he could only lie on his stomach. One night, Ladmaker crawled out of bed to get to the latrine. He was so weak that he fell to the ground sobbing in pain and despair. Levi recounts how his roommate, Charles, reacted:

“Charles lit the lamp…and we were able to ascertain the gravity of the incident. The boy’s bed and the floor were filthy. The smell in the small area was rapidly becoming unsupportable. We had but a minimum supply of water and neither blankets nor straw mattresses to spare. And the poor wretch, suffering from typhus, formed a terrible source of infection, while he certainly could not be left all night to groan and shiver in the cold in the middle of the filth.

Charles climbed down from his bed and dressed in silence. While I held the lamp, he cut all the dirty patches from the straw mattress and the blankets with a knife. He lifted Ladmaker from the ground with the tenderness of a mother, cleaned him as best as possible with straw taken from the mattress and lifted him into the remade bed in the only position in which the unfortunate fellow could lie. He scraped the floor with a scrap of tin plate, diluted a little chloramine and finally spread disinfectant over everything, including himself.” (Gaita, 2004, xvi).

There is no doubt that Charles reacted appropriately. He recognised not only that Ladmaker’s suffering was wrong, but also that, despite his illness, Ladmaker retained his innate worth. Napier rightly says, ‘Loving the afflicted without condescension involves apprehending the inherent worth of the person despite the brumous effects of the person’s sufferings or *low quality of life*’ (emphasis mine) (Napier, 2020, p. 92). In addition, it should be emphasised that a particular mark of love is mercy. Mercy can be understood as an act of personal love that manifests itself in compassion towards a person who has been put in a bad position as a result of experiencing evil (suffering). Love seeks to remove the evil that harms a person (Ferdynus, 2020). While it is not always possible to remove evil, it always becomes possible to show compassion in reliving the danger faced by the sufferer. Love releases what is noblest in humanity: ‘Love is that specific energy which alone allows one to come very close to another person, to enter into his world, and in a certain sense (morally) to identify oneself with his existence’ (Wojtyła, 2017, p. 201). Love makes it possible to recognise in the suffering person their preciousness and irreplaceable value – their dignity.

### Moral Experience

Ethical personalism is expressed in the conviction that the person and their value (dignity) constitute the main norm of morality; that is, the source and criterion of the moral value of an act. At the core of this position is a moral experience that reveals who someone is as a person (Krajewski, 2016, pp. 229–230). Advocates of ethical personalism recognise that moral experience constitutes a source and also direct cognition of the dignity of the person (Szostek, 1995, p. 49). One type of this experience is the personalistic experience to which Wojtyła points.

The person and their value (dignity) is revealed in both external and internal experience. The person is both the subject and the addressee of the action (Krajewski, 2016, p. 232). In the context of external experience – the experience of the person as the possible addressee of the act – Wojtyła formulates a personalistic norm: ‘This personalistic norm, in its negative aspect, states that the person is the kind of good which does not admit of use and cannot be treated as an object of use and as the means to an end. In its positive form the personalistic norm says that the person is a good toward which the only proper and adequate attitude is love’ (Wojtyła, 2001, p. 42). Wojtyła emphasises that, on the one hand, a person differs from other non-personal beings (things) by ‘structure and perfection’ (Wojtyła, 2001, p. 109) and, on the other hand, the proper response to their value (dignity) is love. Wojtyła also recognises that the dignity of the person is revealed above all when the subject experiences the other as a second ‘I’. In this unique experience, an interpersonal relationship is formed in which the other person is recognised as a neighbour. Moreover, this experience allows to experience ‘the other as oneself’ (Wojtyła, 2011, p. 401). Wojtyła maintains that the formation of an authentic interpersonal bond strictly depends on whether ‘I’ and ‘You’ abide in a mutual affirmation of the transcendent value of the person (which can also be described as dignity), confirming it by acts (Wojtyła, 2011, p. 402). Thus, the way to recognise dignity as a transcendent value in others is first to recognise one’s own dignity. On the other hand, the confirmation of the recognition of dignity is an adequate response to this value expressed in acts of love. Both knowing one’s own worth and the worth of others is possible through moral experience – personalistic experience.

Wojtyła’s disciples Styczeń and Szostek seek to clarify the concept of moral experience (personalistic experience). According to them, dignity, which is the object of moral experience, is the source of categorical moral duty (Styczeń & Szostek, 1984). Moral duty not only moves, appeals or shakes the subject out of passivity, but also brings them onto the plane of action. If I only have access to the other as the other ‘I’ through my own ‘I’, then the recognised truth about my ‘I’, about my dignity, reveals its normative power in relation to the dignity of the other ‘I’. What I am not allowed in relation to my own ‘I’, I am not allowed – for the same reason – in relation to any other ‘I’ (Styczeń, 2013, p. 344). Insight into oneself and seeing one’s own dignity allows one to recognise and acknowledge the dignity of others. On this basis, proponents of ethical personalism formulate a principle of conduct: the person of each other is to be affirmed (respected) as one’s own person. In other words, one has a moral duty to affirmation (respect) of the person for their own sake, and this (affirmation) is expressed in acts of love (Krajewski, 2016, p. 235). Thus, moral duty is love as something owed to a person by virtue of the fact that they have dignity.

According to Szostek, the cognition of dignity requires a certain moral maturity. This is because there is no direct ‘proof’ or an ‘external test’ to confirm the belief that each person is precious. However, it seems that every mature and unprejudiced person is capable of perceiving this preciousness (Szostek, 1995, p. 51). To illustrate his intuitions, Szostek refers to the following example:

“Here’s little Kasia playing in the kitchen beating a tureen spoon into a saucepan with all her might. Mum intervenes: Kasia, come on. You make a terrible noise and grandma can’t get any rest, she’s prepared dinner, helped with the cleaning, she’s older now, she’s sick, she wants to get some sleep. And there are times (though not always) when he puts down the saucepan and ladle, tries to keep quiet, still others are silenced. What arguments did the mother refer to? Of course, not directly *to the personalist argument*; one can imagine the bewildered eyes of a child if he were told: *Have regard for the personal dignity of his grandmother and put down the saucepan!* However, let us note an important sense of the mother’s persuasion: she was trying to open Kasia’s eyes to who her grandmother is; to show this truth about her, the respect for which consists in a certain behaviour, not just in a theoretical statement. And the way in which it does so is telling. She tells us about her grandmother: her work, her age, her fatigue – in the hope that little Kasia, through this information, will be able to see who her grandmother is and, therefore, what needs to be done not to hurt her.” (Szostek, 1993, pp. 91–92).

Even if the ways of recognising dignity presented here are not convincing to some participants in the ethics debate, it is hoped that they too will not remain indifferent to certain emotional states and experiences that point to normative references to persons. These include remorse, grief, love and the possibility of directly recognising the preciousness of a person through moral experience.

### Limitations

Given that the search was limited only to some typologies of dignity, additional interpretations of dignity reflecting additional cultural and international perspectives may not have been explored. Based on the findings of the current literature, deciphering the beliefs of dignity internationally is difficult. However, this article may still include the majority of the normative aspects even if it is not exhaustive. Building practical and empirical verifications is the next step that will help identify the possible applications of theoretical philosophical concepts of dignity in health care. In other words, the types of dignity identified in the article can provide theoretical material for their empirical verification in health care practice. Attempts to apply certain types of dignity in nursing practice and clinical care have been reported in the literature (e.g., Gallagher, 2004; Lin & Tsai, 2011; Lindwall & Lohne, 2021). However, additional empirical research is needed to determine, for example, whether and how the types of dignity presented in the article might affect the understanding of dignity by patients, caregivers, family members, or health care professionals. In addition, this submission is a philosophical argument that only tangently relates to health and wellbeing. Future research is required that presents an argument based on empirical evidence about human dignity and health - particularly with respect to issues such as the various bio-psycho-social-spiritual affects of moral injury upon human health and dignity (Carey & Hodgson, 2018). What is more, the types of dignity developed in the article (especially existential dignity and actual and potential dignity) may provide a basis for attempting to redefine ‘dignity’ in various bioethical documents or ethical codes. The issues mentioned here, however, are beyond the scope of this article.

## Conclusions

The purpose of this analysis is to contribute to the understanding of the term ‘dignity’ and the ways of recognising dignity. The scope of the analysis was limited to several typologies of dignity. Some scholars may feel disappointed that they were not included in the analysis or that their views were not presented comprehensively. It should be stressed, however, that the aim of the article is not to present the entire debate on dignity or to provide a comprehensive study of the different types of dignity, but to attempt to outline a map that can be used to identify the different meanings attributed to the term ‘dignity’. More specifically, the analysis aims to formulate a response to the charge of speciesism and to show on what basis it can be claimed that all human beings have dignity. Furthermore, it aims to indicate possible ways of recognising dignity by means of certain emotional states and a specific moral experience developed on the basis of ethical personalism.

It is important to emphasize one more crucial point. The answer to the title question of the article seems clear. Dignity is a necessary moral idea in health care. Dignity cannot be reduced to autonomy, as Macklin and Pinker contend. It need not solely possess a religious character. Referring to the philosophical assumptions of Aquinas and Aristotle, one can reasonably argue that every human being has an immutable and inalienable metaphysical value (dignity) as long as they exist. Furthermore, such dignity can serve as protection against the instrumentalization of every human life and against attempts to strip it of its normative character due to human nature’s defects or a perceived low quality of life. It thus seems that, in light of the views of Aquinas and Aristotle, Macklin and Pinker err in their understanding of dignity and its role in health care.

I realise that the analysis represents only one of many possible proposals for understanding the term ‘dignity’ and for solving the problems signalled in the article. I also realise that the views expressed may meet with both approval and criticism. The article will have served its purpose if it convinces the reader that the understanding of dignity presented in it deserves interest and further debate.
